# Editorial: The tip of the non‐alcoholic fatty liver disease iceberg—the more we know, the smaller it gets?

**DOI:** 10.1002/jgh3.12996

**Published:** 2023-10-30

**Authors:** Sagnik Biswas, Shalimar

**Affiliations:** ^1^ Department of Gastroenterology and Human Nutrition All India Institute of Medical Sciences New Delhi India

Hepatic steatosis (HS) is defined as an intrahepatic fat content of more than 5%.^1^ While the clinical entity of non‐alcoholic fatty liver disease (NAFLD) is based on a diagnosis of intrahepatic steatosis, it was initially considered a benign disease without much clinical significance. A recent meta‐analysis shows that the global pooled prevalence of NAFLD is approximately 30.05%, with the greatest prevalence in Latin America (44.37%).^2^ The meta‐analysis also revealed an alarming rise in global NAFLD prevalence—from 25.26% (1990–2006) to 38.0% in 2016–2019—representing an almost 50.4% increase. Considering that 5–6% of patients with NAFLD progress to NASH, with a further 1–2% progressing to cirrhosis, the absolute number of projected potential cases with liver cirrhosis due to NAFLD is staggering.^3^ It is also crucial to identify the limitations of the studies that provide us with these estimates of global prevalence, as these are mainly hospital‐based studies and suffer from a Berkesonian bias. Thus, the true global prevalence may not be reflected in these analyses and is expected to be higher than what is reported in the current literature.^4^ Thus, in the truest sense, NAFLD is an impending global epidemic.

Another important clinical aspect that adds to the complexity of managing NAFLD is the slow rate of disease progression coupled with the risk of extrahepatic events (cardiac, cerebrovascular, etc.) in this patient group. Indeed, progression from one stage of fibrosis progression may take 14.3 years in patients with NAFLD and 7.1 years in patients with NASH.^5^ Also, the leading cause of mortality in these patients is cardiac and not hepatic.^6^, ^7^, ^8^ Overall mortality and liver‐related mortality increase with the fibrosis stage in patients with NAFLD.^9^ Thus, simple identification of a “fatty liver” based on clinical examination and ultrasound is not enough, and patients should be evaluated to stratify them into risk groups “fibrosis” and provided targeted interventions to mitigate the risk.

New nomenclature has recently been proposed, which includes the terms “metabolic‐dysfunction related fatty liver disease (MAFLD)” and “metabolic‐dysfunction associated steatotic liver disease (MASLD).”^10^, ^11^ The implications of the change in nomenclature extend beyond semantics. The rationale is to introduce “positive” diagnostic criteria and incorporate cardiometabolic risk factors into the diagnosis, which may help in risk stratification.^12^ It is well known that liver fibrosis is the driver of outcomes in patients with NAFLD, and considering the limitations of conventional liver biopsy, there is an emphasis on the assessment of hepatic steatosis using non‐invasive methods—serum biomarkers, elastography, computerized tomography, magnetic resonance imaging, etc.^13^ There is also a need at this time to identify individuals or groups who are at risk of developing NAFLD due to ethnicity or genetic predisposition and initiate lifestyle modification in these groups as a method of primordial prevention.^14^ Rich *et al*. (2018) reported higher rates of NAFLD in the Hispanic population (pooled prevalence 22.9%) in the United States while also reporting lower rates of NAFLD in Blacks (pooled prevalence 13%) as compared with non‐Hispanic Whites (pooled prevalence 14.4%). Even in the presence of risk factors such as diabetes mellitus or obesity, the Black population still had a lower risk of developing NAFLD (RR = 0.85) than the White population.^15^ However, such an analysis is always likely to be limited by the absolute numbers of individuals of each ethnic group included and whether these numbers are representative of the entire group. Due to healthcare disparities, some groups may be marginalized and not adequately represented in these studies. Considering that NAFLD is a lifestyle disorder where the therapy has to be personalized and tailored based on individual patients, it is important to have as much data available on all groups of people as evidently “one size does not fit all in NAFLD.”^16^

To this effect, the study by Thomas *et al*. is focused on two ethnic groups, Pacific Islanders (PI) and Asians residing within the United States.^17^ They retrospectively reviewed computerized tomography images of patients from an integrated healthcare system and provided the results for 748 patients, of whom 295 were Asian and 453 were PI. The Asian representation in the study was predominantly Filipino (62.7%) and Japanese (14.6%), while the Pacific Islanders were predominantly native Hawaiians (74.2%). They found that 41.6% of patients had HS, with no significant differences in prevalence between PI and Asians. HS was similarly distributed between males and females. The odds of having HS were higher in those with higher body mass index (BMI) and those with higher HbA1c values. The study adds to the available literature on NAFLD prevalence and provides data on as‐yet underrepresented ethnic groups. The study also raises several interesting points that could not be addressed due to its retrospective nature and may be considered for future studies.

The role of imaging to identify HS is developing rapidly with the widespread use of computerized tomography (liver attenuation index) and magnetic resonance imaging (proton density fat fraction‐MRI‐PDFF). Multiparametric MRI, which includes MR spectroscopy (MRS), MR elastography (MRE) and T1 mapping, has shown high diagnostic accuracy for NAFLD.^18^ Similar multiparametric CT sequences, which include hepatic attenuation, liver segmental volume ratio, splenic volume, and liver surface nodularity score, have shown good diagnostic ability to identify advanced fibrosis in NAFLD patients.^19^ These newer modalities add to the established diagnostic modalities such as transient elastography and ultrasound and are invaluable in patients with higher BMI (>30 kg/m^2^) where the conventional modalities may provide inaccurate results. However, despite the evident utility of these tests, they require higher technical skill to perform and interpret accurately, which has restricted the availability of these tests to predominantly academic centers. A major limitation of using radiological modalities to diagnose steatosis is that it provides no information on the underlying etiology or associated risk factors. HS is a multifactorial disorder that may be associated with overnutrition (obesity, eating disorders, hypothalamic diseases), undernutrition (protein energy malnutrition), drugs (steroids, atypical antipsychotic agents, anti‐epilepsy drugs), toxins (alcohol), metabolic disorders (diabetes mellitus, Wilson's disease, glycogen storage disorders), etc.^20^ Thus, the imaging evidence of HS alone provides only limited clinical information and should be correlated with clinical history for a complete diagnosis. Diagnosis of NAFLD based on radiological methods alone is an oversimplification and should be avoided.

Another interesting aspect of the study cohort was the diversity of patients in the study cohort. While the absolute numbers of the individual groups were limited, the Asian cohort consisted of Chinese, Vietnamese, Thai, and Korean individuals in addition to the Filipino and Japanese. However, the absence of individuals of South East Asian origin is a limitation to these data as individuals from this region are phenotypically different compared with the Western population. They have greater risks of development of NAFLD and have genetic polymorphisms, which may lead to the development of NAFLD even with normal BMI, which is clinically designated as “lean NAFLD.”^21^ This group of individuals has similar rates of progression of hepatic and extrahepatic disease as compared with those with higher BMI, but there is no effective pharmacotherapy or lifestyle intervention to modify the natural history of disease in this patient group. A recent meta‐analysis from India reported the estimated pooled prevalence to be 38.6% in adults and alarmingly 35.4% in children.^22^ As reported by Draijer *et al*., only one‐third of children with pediatric NAFLD have a resolution of steatosis as adults, while 6% develop NAFLD‐related fibrosis.^23^ These two results highlight the need for increased inclusivity and representation of a South East Asian cohort in studies pertaining to NAFLD.

With increasing globalization and ease of travel worldwide, there has been increased cultural exchange and a breakdown of the traditional concepts used to explain the regional variations in HS, which were reported such as diet, sedentary lifestyle, urbanization, etc. With increased travel, there is increased emigration of individuals from one country to another, leading to exposure to newer risk factors associated with HS. The concept of acculturation, a sociological process in which members of a particular cultural group adopt the beliefs of a different cultural group, has often been cited as a risk factor for NAFLD among individuals who have emigrated. A prior study by Balakrishnan *et al*. reviewing the National Health and Nutrition Evaluation Survey (NHANES) data reported no differences in NAFLD when comparing Mexican‐born and US‐born Mexican Americans, as reported by Thomas *et al*. between Asians and Pacific Islanders. While the data in the current manuscript are cross‐sectional and thus cannot provide additional information like generational status and duration of residence, the study by Balakrishnan *et al*. explored the same but found no significant association with NAFLD. Interestingly, however, they did report that high‐risk factors associated with NAFLD, such as waist–hip circumference, obesity, hypertension, and hypercholesterolemia, were more prevalent in acculturated Mexicans than in less acculturated Mexicans.^24^ Thus, one may assume that changes in diet and exposures in emigrants may lead to the development of risk factors such as obesity (due to changes in dietary constituents and meal patterns), dyslipidemia, insulin resistance, etc., but for the new risk factor to lead to significant hepatic steatosis would probably require a longer time period. Thus, in any study on the influences of ethnicity in NAFLD, the follow‐up should be adequately long, considering the generation of the family from the time of emigration. Another interesting point of study would be to assess whether the children of emigrants who marry native individuals in their new country demonstrate the same risk of NAFLD as their parents or not.

In conclusion, current data on NAFLD are just the tip of the iceberg. Hospital‐based data are easy to collect and analyze but provide us with only a fraction of the true disease burden affecting the society. Recent data have shown that not only affected individuals but even family members of those with MASLD are at risk of developing MASLD and subsequently hepatocellular carcinoma.^25^ In the West, NAFLD and its complications are already the fastest‐growing indication for liver transplantation and threaten to strain the ever‐burgeoning transplant wait list in these countries. Combating this epidemic would thus require concerted efforts from individuals involved at all levels of healthcare, starting from primary healthcare workers, physicians, and epidemiologists all the way up to individuals in charge of designing healthcare programs at regional and national levels.
